# A delayed diagnosis of gastrointestinal foreign body causing reno‐duodenal fistula

**DOI:** 10.1002/bco2.70107

**Published:** 2026-02-24

**Authors:** Hedda Cooper, Jodie McDonald, Shannon McGrath

**Affiliations:** ^1^ Flinders University Adelaide Australia; ^2^ Department of Urology St Vincent's Hospital Fitzroy Melbourne Australia

## 1 DESCRIPTION

**FIGURE 1:**
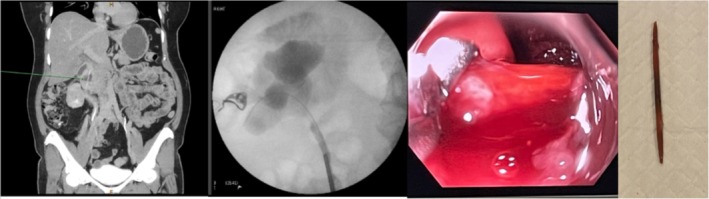
From left to right: (1) computed tomography intravenous pyelogram showing linear foreign body within gastro‐intestinal tract, (2) retrograde pyelogram showing contrast within the duodenum, (3) endoscopic retrieval of toothpick and (4) toothpick upon retrieval.

A 56‐year‐old female presented to our emergency department in July 2023 with right flank pain and fever. Her past medical history included gastro‐oesophageal reflux, hypertension, uterine fibroids and Graves' disease. She had presented to her general practitioner 11 months prior (August 2022) with rapid onset midline low back pain (with no preceding trauma or neurological symptoms) that resolved spontaneously. She had no history of urinary tract infection (UTI). On arrival, she had a urine dipstick (and subsequent microscopy, culture and sensitivity) indicating a UTI, elevated inflammatory markers and a computed tomography kidney, ureter, bladder (CT KUB) showing bulky uterine fibroids thought to be responsible for her significant right‐sided hydronephrosis. She had normal renal function at the time of presentation. She underwent a rigid cystoscopy, retrograde pyelogram (RGP) and insertion of a right ureteric stent. Upon cannulation of her ureteric orifice, frank pus was observed. It was noted that her ureter calibre narrowed at the proximal ureter. Her discharge plan was for hysterectomy to definitively treat uterine fibroids, the presumed cause of her right‐sided hydroureteronephrosis. In October 2023, post‐hysterectomy, her right ureteric stent was removed and noted to be grossly encrusted. Her urine was cultured, showing two species of Candida, which were treated with antifungal medication. She gave a history of recurrent UTIs since her initial presentation in July. She had a follow‐up computed tomography intravenous pyelogram (CT IVP) in December 2023, which showed unchanged hydronephrosis (despite hysterectomy), right‐sided proximal ureteric structuring and a foreign body (reported to be a fishbone by the reporting radiology team) within her gastrointestinal tract (Figure 1). Her renal function remained within normal limits. She underwent a gastroscopy in December 2023, during which moderate gastritis and duodenitis were noted, and no foreign body was visualised. In January 2024, the patient underwent rigid cystoscopy and RGP; during which, contrast was noted in the duodenum (Figure 1), suggesting fistulation caused by the foreign body. The patient underwent a repeat gastroscopy the next day, and the foreign body was removed and found to be a toothpick (not a fish bone as previously reported by the radiology team). The patient was followed up in March 2024 with a rigid cystoscopy + RGP, which showed no further contrast within the duodenum and thus presumed resolution of her fistula. Subsequent urine MCS were negative for UTI, and her renal function remained stable and within normal limits.

It took over 18 months from the onset of this patient's first symptom (back pain; August 2022) until the time of treatment (removal of toothpick, January 2024). While digestive tract foreign bodies are relatively common occurrences, with migration into other organs being rare but dangerous and an important differential to consider.^1^, ^2^ Most (80% to 90%) of foreign bodies pass spontaneously; however, sharp foreign bodies such as toothpicks and fishbones may lead to gastrointestinal perforation in 10% to 15% of cases and have been shown to lead to sepsis, liver abscess, appendicitis and peritonitis.^1^, ^3^, ^4^, ^5^ Unfortunately, there was a six‐month period from the time of presentation to the hospital until eventual diagnosis for our patient, during which she had multiple minor operations and a major operation—a hysterectomy. Throughout this time, she also had multiple computed tomography scans, none of which reported the presence of a foreign body at the time of reporting nor on subsequent review after identification of the foreign body. Her delay in correct diagnosis is likely due to the presence of generalised urinary sepsis symptoms. Additionally, the patient reported no recollection of foreign body ingestion, thus presumed to be a case of accidental ingestion. Additionally, other signs such as haematuria have been noted in prior cases but were not observed in this case.^1^, ^6^ Additionally, back pain is a common sign in other reported cases; however, our patient had a single isolated incident of back pain a year prior that resolved spontaneously and is of unclear significance in this case.^1^, ^6^ When treating patients with urinary sepsis, it is important to consider renal foreign bodies as an alternative diagnosis to ensure patients are treated appropriately and without delay.

## CONFLICT OF INTEREST STATEMENT

The authors declare no conflicts of interest. The corresponding author is not a recipient of a research scholarship. This paper has not been submitted elsewhere. There is one figure included in this paper.
